# Equity in mathematics education

**DOI:** 10.1007/s11858-023-01504-4

**Published:** 2023-06-15

**Authors:** Renuka Vithal, Karin Brodie, Reshma Subbaye

**Affiliations:** 1 University of Fort Hare (Faculty of Education), Alice, Eastern Cape South Africa; 2 University of the Witwatersrand (School of Education), Johannesburg, Gauteng South Africa; 3 University of Fort Hare (Office of the Deputy Vice-Chancellor: Teaching and Learning), East London, Eastern Cape South Africa

**Keywords:** Equity, Diversity, Inclusion, Marginalization, Equity-focused pedagogies

## Abstract

This paper reviews research on equity in mathematics education (excluding gender equity) for the period 2017–2022. From the publications identified, five themes were distilled: conceptualizations and framing of equity in mathematics education; research methodologies and researcher positionalities; equity-focused practices, pedagogies and teacher education; equitable mathematics curriculum content, access and pathways; and equity in mathematics education at system levels, nationally and internationally. The review concludes by engaging some of the critique and suggests future directions for research. The research demonstrates that there is growing voice and visibility of equity-focused studies in mathematics education and that conceptualizations of equity have broadened and deepened through an increasing diversity of studies in this area. At the same time, the review also shows the dominance of the Global North in shaping equity discourses and the paucity of research on equity in mathematics education from the Global South.

## Introduction: Growing voice and visibility of equity in mathematics education

Many societies across the globe are growing increasingly diverse and unequal. Within and across countries, migration due to war, conflicts, climate change, poverty (to name a few), are shifting the nature of contemporary societies. Schools in general and mathematics classrooms in particular, reflect societal diversity and inequities as well as contain the possibilities to transform them. Providing access to quality mathematics education to learners from different cultural perspectives and knowledge backgrounds, diverse racial, ethnic, religious, linguistic, gender, socio-economic status and sexual orientations, is an important endeavor. Within this context, it is not surprising that there has been a growing focus on equity-related research in mathematics education practices, theories, curricula and policies.

In the past five years there have been a number of developments which collectively show this increase in research on equity in mathematics education. In 2022, the Journal for Research in Mathematics Education (JRME) published two special issues on this topic. In their editorial to the first issue, Crespo et al. (2022) show that equity-focused articles in JRME increased from 11% in 2013 to 22% in 2021 and argue that “equity-focused research in mathematics education is now closer to the mainstream of topics with which our field is concerned” (p. 88). In the second issue, a more diverse group of authors commented on the papers in the first issue, leading to a deepening of key concepts (Matthews et al., 2022). ZDM-Mathematics Education commissioning reviews in equity research highlights this growth. Two other leading international journals, Educational Studies in Mathematics and the Journal of Mathematics Teacher Education, have reflected in their editorials on their approaches to equity, and considered how to broaden representation from across the world: both in the national origins of authors and in the conceptualisations of mathematics education in their pages (Brodie, 2022; Mesa & Wagner, 2019; Wagner et al., 2020).

Vithal and Jurdak (2018) note this movement from the margin to the mainstream and argue that conceptions of equity are increasingly being engaged on their own terms or integrated into other areas of research. They demonstrate this through an analysis of the programmes and activities of recent International Congresses of Mathematics Education (ICMEs), where equity has featured more centrally and regularly.

At a system level, data from international studies such as TIMSS have been analysed to make inequities in mathematics achievement more visible, linking these to inequities across classrooms, schools and home backgrounds. These studies have enabled a closer focus on various dimensions of equity in a particular region or country through providing system level perspectives on and evidence of inequities [for example Reddy et al., (2019) on South Africa; Qiu and Leung (2022) on Hong Kong]. Although these international studies have come under criticism for their persistent focus on achievement gaps, this research can and does influence changes in national curriculum policies and resourcing in mathematics education. These studies also demonstrate that although there has been much progress in research on equity in mathematics education, there are still large inequalities within and across countries and that these have real consequences for many of the poorest and most vulnerable learners.

The concept of equity in mathematics education is difficult to pin down. While there are many convergences in what is seen as inequitable, for example, the alienation from mathematics experienced by many marginalized learners, views of mathematics as decontextualized, and the gatekeeping role of mathematics in society (Bakker et al., 2021; Yolcu, 2019), there is also much theoretical and methodological diversity. This diversity occurs because context matters, both in terms of who conducts research and what research is conducted. A very visible divide that became evident in the review is between the Global North and Global South.^1^

This review of research on equity in mathematics education generated five themes, which are used to organize this paper and are explored in the sections that follow. The paper concludes by engaging critique on equity-related studies and suggests future directions for research on equity in mathematics education. This review excludes gender equity since this will be addressed in another review as part of this series of special issues of ZDM-Mathematics Education.

## Conceptualizations and framing of equity in mathematics education

Research on equity in mathematics education draws on a wide variety of theories and frameworks from both within and outside mathematics education. The particular theories selected to underpin particular studies can be linked to the aspect(s) of equity being considered as well as the characteristics of different participants being focussed on in the study.

Several publications in the period under review refer to the theoretical work of Gutierrez (2012), who argues that equity in mathematics education needs to be conceptualised in relation to access, achievement, identity and power, which are interrelated. Access refers to the resources available to support mathematics learning; achievement refers to student outcomes, which are crucial in determining students’ futures; identity refers to the holistic growth of learners as social and cultural beings that mathematics education can and should promote; and power refers to how social relations between different hierarchies play out in mathematics classrooms. Gutierrez notes that the “access-achievement axis” (p. 20) had been dominant in research, leading to what she called “gap-gazing” (p. 31), often informed by deficit perspectives, which focuses on differences in mathematics achievement among marginalised and mainstream learners without a focus on improving the situation. She argues for a stronger focus on identity and power, which she calls the “critical axis” (p. 20), to understand the structural issues involved and possibilities for improvement. Matthews et al. (2022) note that these axes are not orthogonal, they influence each other, for example, access to equitable pedagogies is necessary for identity shifts, which may support stronger achievement.

A number of researchers focussing on social justice in mathematics education in this review have turned outside mathematics education to Fraser’s (2008) framework of how distribution, recognition and representation contribute to normal and abnormal justice. For Fraser, normal justice discussions focus on distribution, without the recognition and representation. Abnormal justice requires participatory parity, i.e.

dismantling institutionalized obstacles that prevent some people from participating on a par with others” (in Meaney, 2022, p. 550)

Meaney et al. (2022) show that abnormal justice discourses in mathematics education conversations occurred when participants made references to aspects of distribution—what kind of mathematics should be taught and learned, recognition—whose cultural practices should be acknowledged as important, and representation—who makes decisions about whom.

Fraser’s and Gutierrez’ frameworks complement each other, with distribution related to the access-achievement axis and recognition and representation related to the identity-power axis. Each framework illuminates which elements of equity are being worked with in particular papers and the extent to which key ideas are being mainstreamed or marginalised. In the papers reviewed from the past 5 years, a strong focus was evident both on aspects of distribution, access and achievement, and on identity, power, recognition and representation.

Conceptions that highlight socio-economic class and other structural differences have featured in equity studies, bringing into sharp relief inequities in mathematics education. Conceptions related to distribution focus on differential mathematics achievement, mathematics as a gatekeeper, mathematics as necessary for social mobility and opportunities available to various communities of students for learning mathematics. These foci continue to be important (Bakker et al., 2021) particularly to highlight how dominant structures, both within and across countries, continue to exclude many students from success in mathematics. From the South African context, Reddy et al. (2019) argue:

The South African challenge, as in other middle-income countries, is to raise the achievement levels of children in schools, decrease the inequalities between the affluent and the poor, and increase the rate of change of progress in achievement outcomes (p. 177)

While Reddy and others note large improvements in poor South African learners’ mathematics achievements in the post-apartheid period (Reddy et al., 2019; van der Berg & Gustafsson, 2019), they also note that the very large inequalities between rich and poor and white and black learners remain.

In the papers reviewed, researchers in equity in mathematics education, have moved to a stronger focus on conceptions of identity and power (Gutierrez, 2012) and recognition and representation (Fraser, 2008). Equity concerns are often combined with concerns for diversity and inclusion in mathematics classrooms, and with a focus on building social and racial justice (Bakker et al., 2021). As Valoyes-Chavez and Darragh (2022) note, there has been progress:

in centring issues of equity, social justice, dignity, and respect in mathematics education research, policy, and practice. At this point, for any researcher in the field to deny the racist, sexist, ableist, classist, and patriarchal nature of the mathematical education system of practices is becoming more difficult (p. 375).

The focus on race and racism has led to several theories being used in studies of equity in mathematics education. Joseph et al. (2019) argue for two key concepts in understanding a more humane conception of pedagogy for black girls: social interaction, which refers to teachers and learners building identity and community together; and power sharing, which supports collaborative inquiry in mathematics, valuing and working with all learners’ mathematical contributions.

Theories related to (de)colonisation have been invoked in studies that attend to issues of equity for indigenous participants in mathematics education. Allen and Trinick (2021), writing about mathematics education for Maori students in New Zealand, argue that the maldistribution of resources due to colonization, is key to understanding the marginalisation of indigenous people in mathematics. Access is an important indicator of the problem of inequity, and together with achievement, reveals structural inequalities.

Conceptions of equity reflect who are imagined as students in mathematics classrooms. Recent research on neurodiversity and disability shows that teachers significantly underestimate the mathematical capabilities of disabled students (Hunt et al., 2022; Lambert & Tan, 2020). Medical models of disability reflect deficit perspectives, while social models argue that disability is context-specific and produced interactionally (Lambert & Tan, 2020).

More recent theoretical work has focussed on new aspects such as the emotions and emotional labour involved in learning mathematics and overcoming oppression (Valoyez-Chavez & Darragh, 2022). A key element of emotional work is care (Brodie, 2017; Watson, 2021). Watson (2021) argues for care in three dimensions: care for mathematics, care for learners and care for learning mathematics, developed through collaborative inquiry and power-sharing among teachers, learners and communities. Listening and noticing are important for both the emotional and cognitive work involved in learning mathematics. Van Es et al. (2022) developed a framework for teachers’ multidimensional noticing, which includes explicit attention to students’ sociocultural selves, and how they position themselves and others in the classroom, that is, how they represent themselves and are recognised (Fraser, 2008).

In elaborating conceptions related to recognition and representation, three related conceptions of equity, diversity and inclusion were identified in the review as important areas of current and future research: respect for the humanity and dignity of marginalised learners; emotions and emotional labour in mathematics classrooms, as important aspects of identity; and broadening who needs to be included—thus expanding research to issues of neurodiversity, immigration, and gender fluidity, in addition to previous categories of poverty, race and gender.

In summary, conceptions of equity over the past 5 years have expanded. There is more work on recognition and representation of learners from marginalized communities, framed by and developing further our understandings of power, participation, identity, emotions and human dignity. There is also continued work, predominantly based on international studies on monitoring access to and achievement in mathematics. The former conceptions of equity predominate in the Global North, the latter in the Global South.

## Research methodologies and researcher positionalities

The wide diversity of research on equity in mathematics education, with different conceptions and theorising of equity, involving different student and teacher participants, in a variety of contexts, using many different research methodologies and addressing many different questions on the topic, demonstrates the complexity of this area of research, and the appropriateness of a narrative review. Narrative reviews, commonly found in the literature of various disciplines, offer critical analyses of the literature on a topic, theme or concept. Narrative reviews are useful to get an overview of a topic, for policy makers and researchers to identify gaps and for practitioners seeking reliable, and current information (Byrne, 2016).

In searching for papers published in the period 2017–2022, for this review, academic journals on mathematics education were identified using Google Scholar metrics (n = 20), ERIC (n = 6) and ScienceDirect (n = 1). Each of the 27 journals were searched using the terms equit*, diversity and inclusion. The search was then expanded to non-mathematics education journals, identified using Google Scholar and using the search terms: mathematics and education and equit*. These two searches yielded 82 journal articles. Two further searches were conducted of websites of international studies of TIMSS and the World Bank, as well as selected mathematics education conference websites (e.g. Southern African Association for Research in Mathematics, Science and Technology Education, ICMI Study 24). In total, all four searches yielded 102 publications. These publications were screened for relevance to the topic. In addition, well-known international scholars in this area of research (18) were requested to recommend one key publication (8 responses received). The literature selected for this narrative review is limited to publications written in English and excluded publications on gender equity (as per the brief for the review). From the publications on equity in mathematics education that were reviewed, 58 are referenced in this paper. For this narrative review, a thematic coding framework guided the review process.

A review of methodologies showed two main kinds of research on equity in mathematics education. First, the majority, are smaller scale studies focusing on individuals or groups of teachers, students, classrooms or schools to examine issues of equity or interventions to achieve equity in mathematics education, which are mainly qualitative. Second, are the large scale national or international studies, which are mainly quantitative focusing on mathematics learning outcomes and related to a range of variables on the conditions and resources for teaching and learning at different levels of education systems.

The first group of studies use methods such as classroom observations (e.g. Louie, 2017; Walkington & Marder, 2018), interviews (e.g. Hunter et al., 2020; Yilmaz et al., 2021) and focus group discussions (e.g. Tremain et al., 2022). These sources of evidence were collected locally and used to explore or explain issues of equity in relation to mathematics classroom practices, student experiences and curriculum and assessment choices.

The second group of studies include mainly surveys (Reddy et al., 2019; Xie et al., 2021), which are used to describe and compare various demographic distributions in mathematics learning outcomes and inequities in distribution of resources and conditions for learning. The quantitative studies often draw from large national and international data sets to provide insights about global patterns and trends and are also increasingly analyzed to focus on a particular country or region (Qui & Leung, 2022; Reddy et al., 2019). While these studies provide insights into patterns of inequality, they do not offer insights about the subjective experiences of inequality.

Some studies use mixed method approaches and case studies combining observational tools, interview data, discourse analysis and survey data (Hunt et al., 2021; Schnell & Prediger, 2017; Louie, 2017; Walkington & Marder, 2018; Semana & Santos, 2018). Only a few papers explored the affordances and limitations of the choice of methodological approach in equity-related research (for example Reinholz & Shah, 2018; Walkington & Marder, 2018).

The question of who conducts equity-focused research, and researchers’ understandings of how their own positionality and identities might reproduce or disrupt existing power dynamics in the study context, are important. This requires reflecting on the bias inherent in shaping how a study unfolds and is contextualized, and what meanings and insights are privileged. Some qualitative studies explicitly declare the researcher’s positionality in relation to the study participants (Battey et al., 2022; Matthews et al., 2021) but only a few interrogate the researchers’ relationships with research participants (Wright, 2021). Wright (2021) who reports on a participatory action research project which aims to transform traditional pedagogies, shows how the researcher’s positionality and critical reflection on the research process, can transform classroom practice.

Notably, most of the researchers studying equity in mathematics education are in the Global North. The dominance of this geographical positioning may go some way in explaining why certain conceptions of equity and approaches to research on equity in mathematics education are foregrounded. It could be speculated that issues of equity in Africa for example (which are largely absent in this review), where inequities from poverty, unemployment and rurality dominate, could generate different research questions and approaches.

## Equity-focused practices, pedagogies and teacher education

The biggest subset of the literature identified was in the area of equitable mathematics education practices and pedagogies. This work reflects the increased focus on relationships among access, identity and power as discussed earlier, particularly the imperative to research and act with key participants such as teachers and students in addressing inequities. In this section the focus is on practices, pedagogies, teacher education and the impact of the recent COVID-19 pandemic on equity in mathematics education.

A large number of equity-focused studies investigate what mathematics teachers think, say and do (see reviews by Civil et al., 2019; Yolcu, 2019; Roos, 2019). Shifts in teachers’ views and practices toward student-centred approaches (e.g. Felton-Koestler, 2019) or away from ability groupings (Hunter et al., 2020, Hunt et al., 2022) are some examples of studies on teacher practices. How teachers talk to learners is among these. Vogler et al. (2018) show that students of high and low socio-economic status are differentially attentive to the teacher’s contextual expectations. Deficit talk in classrooms, which systematically devalues marginalised students and/or their families based on perceived deficits of their mathematics achievement or “ability” are still widespread in mathematics classrooms (Byun, 2022; Louie, 2017). Newer teacher-noticing practices (Schnell & Prediger, 2017; van Es et al., 2022) have been studied to promote equity in mathematics education by developing teachers’ attention to the resources and strengths that students bring to their mathematics learning and acknowledging the diversity of mathematical thinking among their students.

A number of studies that focus on teaching, demonstrate a diverse range of equity-supporting pedagogies, including: culturally responsive mathematics pedagogy, using mathematics to interrogate issues such as food security (Ramsay-Jordan, 2021); teaching for active learning aligned with an inquiry approach, where students inquire into mathematics and teachers inquire into students’ mathematical ideas (Tang et al., 2017); pedagogies for mixed ability groupings, which support a more expansive view of students’ mathematical capabilities (Hunter et al., 2020; Meyer & Slater-Brown, 2022); pedagogies that integrate mathematical activities with language and culture (Nortvedt & Wiese, 2020); and rehumanizing pedagogies (Joseph et al., 2019). Different students may require different pedagogies, for example, disabled students (Hunt et al., 2022), black girls (Joseph et al., 2019) or migrant students (Nortvedt & Wiese, 2020).

The literature also demonstrates the difficulty of making equity-supportive changes in pedagogy. For example, Meyer and Slater-Brown (2022), in their study of mathematics lead teachers’ incorporation of mixed ability grouping into school-wide practices, found that only two of six lead teachers were able to make changes to their own practice and only one was able to implement school-wide practices. Gardee (2019) shows how, while some teachers offer identities of affiliation to learners, supporting their participation in mathematics classrooms, others offer identities of marginalization, which serve to exclude learners from mathematics, thereby pointing to the importance of both social and pedagogical relationships between teachers and learners in supporting learners’ mathematics identities.

To support more wide-ranging changes towards equitable practices and pedagogies, learning about equity has been incorporated into mathematics teacher education programs for both pre- and in-service teachers to become change agents. One issue that arises is how narrowly or widely equity issues should be engaged within programs. Mintos et al. (2019) found that mathematics-specific equity learning opportunities in secondary pre-service programs were most frequently related to issues of access and achievement in mathematics, while issues of power and identity were more often discussed in the general courses, although these were related to each other in the program. Another issue is how to attend adequately to issues of equity involving different groups of students, particularly in relation to which pedagogies might be more generic, and which need to be specific to different students. For example, Tan and Thorius (2019) document how a professional learning community of educators employed an equity expansive learning frame to support disabled learners to engage in higher level mathematics inquiry.

The period of this review includes the major global phenomenon of the COVID-19 pandemic and the shift to online mathematics education. Several publications focus on the implications of inequitable digital resourcing and on the rapid changes that had to be made in teacher practices and pedagogies. Teachers’ beliefs, expectations for students, access to resources, and students’ socio-economics status were all found to be relevant factors in supporting or constraining equity during the pandemic (Yilmaz et al., 2021). Although skilled and caring teachers leveraged prior experiences, innovative methods, collegial support and technological tools to support students and families (Ruef et al., 2022), distributing digital resources for supporting online mathematics education for under-resourced communities was especially challenging (Allen & Trinick, 2021). Remote instruction widened inequalities in mathematics learning (Yilmaz et al., 2021). Uegatani et al. (2021) reported changes in students’ identities in mathematics learning and lost opportunities to receive positive feedback and to learn the social aspects of problem solving. Contrary to many negative impacts recorded, a study by Xie et al. (2021) on online micro-classes for primary mathematics in response to the COVID-19 pandemic, found high student approval levels for the intervention, which did not differ across gender, socio-economic status, school location and previous achievement, thus promoting digital equity. The pandemic perpetuated many inequalities while showing some possibilities for building equity going forward.

In summary it is evident that a broad and diverse range of practices and pedagogies in mathematics education have been researched with explicit reference to equity. One key observation is that in most instances only partial successes have been found. This is aptly demonstrated in the study by Louie (2017) who found that even for teachers who express a strong commitment to equity, and participated in ongoing equity-oriented professional development, the dominant culture characterizing mathematics education—a culture of exclusion—persisted. So considerable challenges remain in achieving equitable pedagogical practices in mathematics education.

Despite there being a large number of studies in this area, synthesizing and finding common threads to discern clear guidelines for successful equity-related practices or pedagogies presented a challenge, due to: the wide diversity of contexts and conditions studied; the diverse theoretical framings; varied research questions and research participants; the dominance of small-scale studies; and because where success was achieved, it was limited and qualified.

## Mathematics curricula, content, access and pathways

The assertion that mathematics curricula are not neutral, objective or value-free has long been argued in the mathematics education literature (Yolcu, 2019). Issues of equity arise when access to mathematics as a subject or to different content topics within mathematics is enabled for some groups of students and not others, and which then has consequences for their future education, career and life opportunities.

A key example is how precalculus and calculus in the USA context have been shown to limit minority students’ access to particular mathematics pathways (Battey et al., 2022; Bressoud, 2021; Tremaine et al., 2022). These are deemed gatekeeper courses (Battey et al., 2022) since performance in these determine access from schooling into particular college and university courses. Not all schools offer advanced calculus courses, so the more privileged students have the greatest access and the inequality continues into higher education, because few universities attend to disparities in student preparedness for university curricula (Bressoud, 2021).

The situation of the USA described above is relevant in many other contexts in which the high school curricula track students into different pathways, and in which access and progression in mathematics is deeply implicated. For example, in South Africa, students must choose between mathematics or mathematical literacy from grade 10. While mathematics is important for students wanting to pursue careers in STEM fields, mathematical literacy is about applications of mathematics and critical citizenship, for example making sense of national debates on crime where quantitative arguments are used (Volmink, 2018). Both mathematics and mathematical literacy are important for all students but students from lower socioeconomic backgrounds, are disproportionately overrepresented in mathematical literacy, which significantly limits their opportunity to gain access into a wide range of higher education programs and careers. Similarly, in Australia, Murphy (2019) found that nonmetropolitan schools in Victoria Australia, that enroll mainly disadvantaged students, are less likely to offer advanced mathematics and their students are less likely to choose those options.

A key point arising from these studies is the marginalizing impact of mathematics curricula for certain groups of students. Who can access and participate in particular mathematics curricula and who succeeds or fails has major material consequences for students’ trajectories through the education system and for their future lives. Equity issues arise more sharply in mathematics curricula because their impact is experienced beyond the learning or mastery of particular mathematics content. Tabron et al. (2021) point to how detracking must go beyond simply providing access to mathematics, to developing and having an equity orientation, which includes having a historical understanding of past policies and practices that have led to the inequities in the current context. A framework for increasing access and achievement of minority and marginalized groups is presented by Tremaine et al. (2022) who argue for recognizing the identities and powers of such groups showing that students are not passive but active learners with agency who can and do overcome inequities.

In the literature from the past five years there was limited research on culturally relevant mathematics curricula linked to equity but a greater focus on equity-related culturally responsive teaching practices and pedagogies (as discussed earlier), with such studies including elements of culturally-relevant curricula. For example, culturally relevant curricula can support inquiry learning, with tasks in which students create and solve their own problems (Tang et al., 2017); or use mathematics to interrogate current social, political and cultural issues (Ramsay-Jordan, 2021). The dilemmas of developing comprehensive culturally-relevant curricula are described by Allen and Trinick (2021) in referring to the Maori-medium mathematics curriculum in New Zealand, where not only the language, but many key concepts needed to change. A culturally relevant Maori curriculum could not easily mirror the standard English curriculum. Taken together, culturally relevant curricula and culturally responsive pedagogies are important to make space for students’ lived experiences, meanings and identities in mathematic classrooms (Yilmaz et al., 2021; Allen & Trinick, 2021).

Official mathematics curriculum documents often reference equity outcomes or goals directly or indirectly. However, when inclusive mathematics curriculum reforms are put in place, thought is not often given to what marginalized learners and teachers need, in order to benefit equitably from the reforms (Karsenty, 2018; Oteiza, 2018). Bartell et al. (2017) provide a framework which they argue will advance future research-based equitable mathematics practices and make achieving high curriculum standards a possibility for all students. Moving forward, it is important to understand how mathematics curricula changes at a policy level can drive equity approaches as well as research on equity in mathematics education.

## Equity in mathematics education at system level

At global and system levels, international studies and reports provide useful analyses that reveal multiple inequities within and across countries and may support policies for equity. One of the best known international studies on mathematics achievement is TIMSS, which has in recent years made it possible to examine inequities more broadly by expanding their focus to a diversity of contextual and environmental indicators and measures to better understand and explain differential mathematics learning outcomes (Mullis et al., 2020). Data from these studies have been analyzed further by researchers from different countries participating in these global studies.

Qiu and Leung (2022) draw on TIMSS data (2011–2019) to show the effects of various student-, family- and school- level variables on mathematics achievement in Hong Kong, which has performed very well in TIMSS. They found not only that family socio-economic status had a significant impact on mathematics achievement but that this was increasing while school location (urban–rural) and school resources were found not to have significant effects. By contrast, in South Africa, school location and resources have been associated with mathematics outcomes. Reddy et al. (2019), analysing TIMSS data (1995–2015), showed how mathematics achievement and contextual inequality gaps are linked in South Africa, which is consistently among the worst performing countries in TIMSS. While observing significant overall improvements in mathematics achievement, the severe consequences of societal inequalities in South Africa are evident in the finding that the achievement gap between learners attending no-fee (poorer) and fee-paying (better off) schools was one standard deviation, with learners in fee-paying schools having access to higher levels of resources and home educational activities which continue into the school environment. Also analyzing South African TIMMS data, Arends et al. (2021) found that academic achievement was significantly associated with economic capital (school resources) and social capital (school climate). Their results show that well-functioning school contexts, free of violence, provide better opportunities for students to succeed in mathematics; and that placing more emphasis on the use of resources and school climate, significantly reduced the variations between schools.

International studies, which generate media attention on mathematics education in particular countries, do sometimes put the spotlight on inequalities and lead to changes in mathematics curricula, teacher education and resourcing. Notwithstanding critiques of such studies, Nortvedt (2018) explains how the publication of PISA results in Norway showed stable achievement differences over time between immigrant and non-immigrant students and led to changes in their quality systems and testing, school mathematics curricula, mathematics teacher-education and a focus on the high number of low achievers in mathematics. International studies can also be used to validate existing policy directions. In South Africa, the TIMSS results, which often make media headlines, have enabled analyses which make inequalities visible between and within school categories at a system level and provided the educational and political justification for various kinds of interventions to be prioritized (Arends et al., 2021), which may in part, explain the overall system level improvements in mathematics achievement, albeit from a low base (Reddy et al., 2019).

The work discussed above suggests that a focus on monitoring achievement gaps and the structural reasons for them, is still important, even in wealthy countries, as long as these present a starting point for thinking about appropriate interventions. In contexts in which achievements in mathematics are very low, in low- income countries, in countries where large proportions of a particular student group are not taking mathematics or are performing poorly in mathematics content that counts for access into high status STEM careers, these international studies offer a means to bring inequities in mathematics performance as well as in conditions for learning into sharp relief and provide a means to lobby policymakers, politicians and government officials responsible for education and the allocation of resources. This is especially the case in contexts where resources for undertaking such studies and the research expertise and capacity may be limited. This point is relevant to partially address Matthews’ et al. (2022, p. 347) call to “ask how we can cultivate a more inclusive discussion of what is necessary, good, and just for differentially situated international communities and spaces”.

International studies provide a window into the Global South context that is visibly underrepresented in the mathematics education literature in general, and on issues of equity in particular. To offer a perspective on this absence, this review includes the concept of Learning Poverty, which refers to a child not being able to read and understand a simple text by age 10 (World Bank, 2022). While this concept can be critiqued, it is a critical indicator of inequality since a child who cannot read proficiently, is unlikely to be able to learn mathematics or any other subject, and is significantly disempowered. Their most recent data estimates that the global Learning Poverty has risen sharply to 70%, and as high as 90% for sub-Saharan Africa following the COVID 19 pandemic. The report notes that “globally, between February 2020 and February 2022, education systems were fully closed for in-person schooling for about 141 days on average. In South Asia and Latin America and the Caribbean, children lost on average 273 and 225 full days of school, respectively” (p. 7–8). While the studies reported earlier attest to the challenges that have been experienced by better resourced countries in the COVID-19 period to provide an equitable mathematics education, it is necessary to draw on all available conceptual and other resources to fully understand the effects of the pandemic and the perpetuation of inequalities across all countries.

## Conclusion: critique and future research

In summary, this narrative review of research on equity in mathematics education published in the period 2017–2022 was generative of five themes, which are interlinked. The two themes on theories and research methodologies shows how much this work has expanded and how diverse it has become. Within the overarching fifth theme examining equity in mathematics education at a system level, whether nationally or internationally, are the third and fourth themes. The third theme, represented by the largest volume of such studies in this review period, demonstrates the many explorations in mathematics practices and pedagogies, including in teacher education, taking place within classrooms and schools to focus on all kinds of inequities and innovative ways by teachers and researchers to address these. The fourth theme on mathematics curricula content, access and pathways places the student in the centre of this research review, drawing attention to how the mathematics education journeys of individuals or particular groups of learners are impacted through the system to eventual careers and job opportunities or to being lost from the mathematics education system.

The reviewed research demonstrates the complexity of studying equity in mathematics education, which requires a close focus on students and teachers in mathematics classrooms and schools while simultaneously remaining connected to inequalities more broadly. The range of conceptions of equity in mathematics education and the diversity of findings are manifest in the difficulty of circumscribing and selecting literature for a review of this area of research.

The literature on equity in mathematics education highlights several tensions and contradictions that need to be embraced and engaged. On the one hand, focusing on equity can preserve the margin and marginal categories, while on the other hand, not focusing on equity and calling out these inequalities, limits the opportunities to make visible or act on the inequalities to improve the situation for those who are most negatively impacted by discriminatory practices and policies.

Any research on equity in mathematics education captures a particular historical moment. For example, race and racism in mathematics education have come into sharp relief in the USA in the current political period and are generating wider and deeper scholarship in this area. Matthews et al. (2021) draw attention to mathematics education for Black communities and the need for reimagining all aspects of mathematics teaching and learning, knowledge and experience in advocating for social justice. There is however, a critique of this very focus on marginalized or disadvantaged students, argued by Martin (2019), in that equity-oriented discourses in mathematics have served to retain the status quo and kept Black learners contained in the very same relative positions. He suggests, as a first step, the notion of refusal as a strategy for such learners to resist the “anti-Black character of mathematics education” (p. 459).

This review reveals that even in critique context matters. The issue of race and racism in contexts that involve minority groups might be different in key aspects compared to a context where the group experiencing discrimination is in the majority (for example the case of South Africa). Notions of power (political or economic) and identity have some invariant aspects but may be characterized or expressed in quite different ways in schools and mathematics classrooms in different contexts.

In much of the literature that we have reviewed, advocacy for equity is foregrounded, together with a strong agency to address marginalization, discrimination and to advance social justice in mathematics education so that as many students who wish to learn mathematics are able to access it and to do so and succeed. While research on equity in mathematics education is focused on marginalized students, with analyses of what has been done to attempt to deal with inequities, more studies are needed that construct such students as active learners, and that research student agency in overcoming inequities, giving their voices greater visibility. Further, a focus on intersectionality in equity studies is needed, that is, studying the intersections among various categories of marginality in mathematics education. More controversially, equity-focused studies need to research privilege, in order to better understand the mechanisms at work, and to support action on addressing inequities for the marginalized. Even as the critique continues, it has the potential to inspire agency and action across contexts in research, policy, theories and practice by thoughtfully acknowledging that what works in one context at one moment in time may or may not do so in another.

Among equity scholars it is well-known that researchers, reviewers and readers of research on equity in mathematics education are never neutral nor value-free. We write and read ourselves—our identities and subjectivities—into the text. Hence, we acknowledge our own positionalities, as three women researchers in South Africa, in a continent that is largely absent in this review of equity in mathematics education. Our authorship of this text reflects our perspectives of being from the Global South, and our histories of colonialism and apartheid in acknowledging the lens through which we make some aspects visible, what we attend to and what we exclude when we reviewed the literature on equity in mathematics education. We have been conscious of the tensions throughout the process of this review, acknowledging the tremendous work done in the Global North, and valuing this work, while being concerned that various structural conditions maintain the marginalization and silence of Global South researchers.

It is outside the scope of this paper to engage a substantial discussion on the challenge of supporting more research on equity in mathematics education in the Global South where inequities involve large sections of society, and where issues of survival loom large in the face of inequalities such as severe poverty and climate change. We have speculated earlier that research focusing on the Global South may require asking different questions, and alternate theories and methodologies that speak to inequities in these contexts. This will require researchers with the vision and courage to approach research on equity in mathematics education from different perspectives and framings of equity.

## Footnotes

1 The terms Global North and Global South distinguish among countries along the lines of socio-economic and political characteristics. Global North countries have stronger economies, infrastructure and technology, while Global South countries have less diverse economies, are characterized by poverty and inequality, and have often had a history of colonization by countries in the Global North. Global North countries are usually in Europe, North America, Australasia and some parts of Asia, while Global South countries are predominantly in Central and South America, Africa and some parts of Asia.
